# Insights from a National Survey on Controlled Substance Diversion Practices in U.S. Hospital Pharmacies: Opportunities for Enhanced Surveillance and Compliance

**DOI:** 10.3390/pharmacy12060183

**Published:** 2024-12-04

**Authors:** Samantha S. Bastow, Eric P. Borrelli, Julia D. Lucaci, Heather Nelkin, April Graves, Amanda Hays

**Affiliations:** 1 Department of Medical Affairs, Becton Dickinson and Company, Franklin Lakes, NJ 07417, USA; heather.nelkin@bd.com (H.N.); april.graves@bd.com (A.G.); amanda.hays@bd.com (A.H.); 2 Department of Health Economic and Outcomes Research, Becton Dickinson and Company, Franklin Lakes, NJ 07417, USA; eric.borrelli@bd.com (E.P.B.);

**Keywords:** diversion, controlled substances, patient safety, hospital pharmacy, healthcare safety

## Abstract

This study explored controlled substance (CS) diversion surveillance practices within hospital pharmacies across the United States. A survey with questions based on published CS diversion risk points was conducted in May 2024. A total of 66 participants from 31 states responded, with 54.5% from single facilities and the remaining from health systems. Most respondents were pharmacy directors, managers, or those in dedicated drug diversion roles. Over 70% have dedicated surveillance teams and use drug diversion software. Results highlight variation in practices, with larger institutions generally showing better compliance. Compliance in procurement and receiving was high for access measures; however, auditing of processes was lower. The lowest procurement compliance was in monitoring periodic automatic replacement (PAR) levels and validating orders with wholesalers. Storage practices showed high compliance in deploying cameras, but low compliance in monitoring them. Dispensing practices had high compliance for restricting CS in automated dispensing cabinets, but low incidence of witness verification during stocking. Waste and disposal practices were well-followed, but training on detecting potential signs of medication tampering was less common. The survey highlights that while strategies to prevent CS diversion exist, their implementation varies. Enhancing monitoring, auditing, and training is essential to strengthen diversion prevention efforts in hospital pharmacies.

## 1. Introduction

Diversion of controlled substances (CS) in healthcare facilities is not rare and is often underreported [1,2,3]. Healthcare organizations bear the responsibility for the development and implementation of programs aimed at preventing CS diversion, ensuring the program adheres to relevant state and federal laws and regulations [3]. According to the Drug Enforcement Agency (DEA), the registrant holds the responsibility for the secure management of controlled substances during the entire medication use process [4]. They are also responsible for employing technology to improve monitoring and control, aiding in the prevention and detection of potential diversion [3]. In recent years, there has been an increased awareness of diversion and many healthcare organizations have come under scrutiny for their ineffective processes for CS management [1,5,6]. Diversion can lead to serious safety hazards for both patients and healthcare workers, inflict reputational harm, and result in significant financial burdens for healthcare systems [6,7,8,9,10,11,12,13,14,15,16,17].

Historically, drug diversion programs have focused on detection strategies in hospital patient care areas, particularly those deemed as high-volume and high-risk based on variable workflows (e.g., Emergency Departments, Operating Rooms, Procedural Areas). The 2022 American Society of Health-System Pharmacists (ASHP) Guideline on Preventing Diversion of Controlled Substances also names the hospital pharmacy as a high-risk area [3]. The pharmacy faces unique risk due to the substantial quantity of CS medications it handles across common risk areas including procurement and storage, preparation and dispensing (including automated dispensing cabinet management), and waste, removal, and destruction [3,18].

A survey of pharmacy directors revealed a considerable variation in the use of recommended practices for preventing and detecting controlled substance diversion with larger institutions being more likely to implement recommended practices compared to smaller ones [19]. The recommended safeguards to prevent CS diversion in this space include careful configuration of healthcare technologies and proactive system-based strategies to mitigate diversion risks [18]. It is important to “enable processes in the pharmacy that enforce documentation and traceability of controlled-drug inventory and all who have accessed it” [18]. In addition, two published studies have also evaluated and outlined possible failure modes for drug diversion within the hospital pharmacy [18,20]. These studies demonstrate risk points across all stages of the medication use process, including CS storage, prescribing, preparation, dispensing, administration, waste, returning, and disposal. In the analysis by deVries et al., 220 failure modes were identified in CS handling within the hospital pharmacy, of which roughly 15% were considered critical failure modes for CS diversion risk [20]. Notably, three main categories of failure modes were delineated—CS handling, data entry, and verification. The limited scope (two practice sites) of these studies highlights a gap in generalizable data to address the current state of diversion surveillance in the hospital pharmacy. Further, the lack of data underscores the need to raise awareness and identify strategic opportunities for health systems to address diversion. The purpose of this study was to gain broader insights into the current CS diversion surveillance practices within hospital pharmacies across the United States.

## 2. Materials and Methods

A Qualtrics survey (Silver Lake; Seattle, WA, USA) was disseminated through the International Healthcare Facilities Diversion Association (IHFDA) membership listserv for a three-week period in May 2024. The survey instrument was developed through a multi-step process involving a thorough literature review of best practices in CS diversion surveillance, and consultation with former hospital pharmacy directors. The survey questions were developed from published studies that outlined CS diversion risk points within a hospital pharmacy using failure mode and effect analysis [18,20]. Questions were also designed to align with the 2022 ASHP guidelines for best practices in drug diversion prevention and surveillance [3]. The complete list of survey questions are available in Appendix A.

All survey responses were included unless they met any of the following exclusion criteria: incomplete responses (defined as less than 90% of fields completed), respondents indicated they did not perform drug diversion surveillance activities, and respondents who declined participation.

The survey was divided into the following medication management categories: procurement, receiving, storage, packaging/compounding, dispensing, and waste/return/disposal of controlled substance medications. Survey responses were analyzed based on whether the respondents indicated they held a system-level versus facility-level role in drug diversion surveillance activities.

Efforts were made to mitigate potential response bias by ensuring that the sample included a diverse range of hospitals based on size, geographic region, and hospital type. A comparison of respondent demographics with national benchmarks was completed to confirm that the sample is representative of the broader hospital pharmacy population [21].

The WIRB-Copernicus Group (WCG) Institutional Review Board (IRB) approved this study and found this research meets the requirements for a waiver of documentation of consent.

## 3. Results

Of 482 users in the IHFDA listserv, we received 101 responses. Of those 101 responses, 35 were excluded; 80% of excluded surveys were due to incomplete responses (defined as less than 90% of fields complete). Other reasons for exclusion included respondents indicating they did not perform drug diversion surveillance activities (17%) and those who declined participation (3%).

### 3.1. Demographics

Of the 66 responses included in the results, 54.5% represented a single facility, and the remaining 45.5% represented a system of facilities (Table 1). Among the single facility respondents, the largest representation was community hospitals (39.4%), followed by academic medical centers (16.7%), government hospitals (5.6%), long-term care facilities (2.8%), and surgical hospitals (2.8%). Additionally, 50% of facilities reported a bed size of less than 150, 25% reported 150–300 beds, and 22.2% reported 301–500; only one facility reported a bed size of greater than 500.

The responses represented 31 US states from the following geographic regions: South Atlantic made up 24.2%, followed by the East North Central (19.7%), West South Central (10.6%), West North Central (9.1%), Pacific (9.1%), New England (7.6%), Middle Atlantic (6.1%), East South Central (6.1%), and Mountain (4.5%). The majority of respondents were Directors of Pharmacy (37.9%) followed by Drug Diversion Lead/Director (28.8%), Pharmacy Managers/Supervisors (16.7%), and Drug Diversion Team Members (10.6%).

The comparison to national data indicated that the sample was broadly representative of U.S. hospital pharmacies, with similar distributions in terms of facility size, region, and pharmacy roles. Lastly, 71.2% of respondents indicated they utilize drug diversion software, and the same number indicated they have a dedicated drug diversion surveillance team.

### 3.2. Controlled Substance Procurement and Receiving

For responses from both health systems and single facilities, the most common team member who authorized purchase orders for controlled substances was the Pharmacy Manager (86.7% and 80.6% for health systems and single facility, respectively) (Table 2). The next most common was the Pharmacy Buyer (50.0% and 55.6%) and Pharmacist Non-Manager (40.0% and 33.3%). One notable difference was that 11.1% of single facilities reported Pharmacy Directors performing this duty, whereas this was found to be zero for Health System responses.

**Figure 1:**
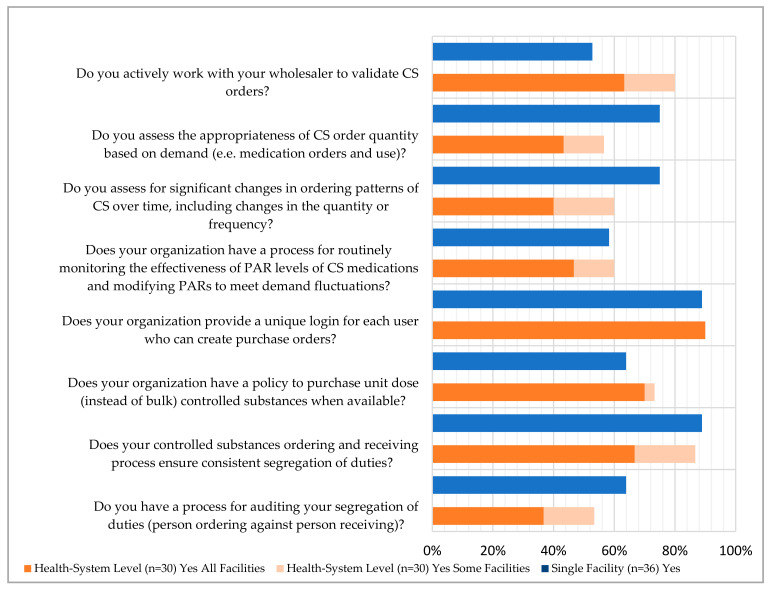
Procurement and Receiving Survey Responses.

For the eight questions regarding the process for procurement and receiving, over half of single facilities and health systems responded they were meeting the current published recommendations (indicated as a “yes” for a single facility or “yes for all facilities” or “yes for some facilities” for health systems) (Figure 1). The highest reported compliance was for organizations providing a unique login for each user who creates purchase orders (approximately 90% for both respondent types). Reported compliance for using consistent segregation of duties for ordering and receiving processes was 86.7% and 88.9% for health systems and single facilities, respectively. When asked about whether there is an auditing process for those segregations of duties, 53.4% and 63.9% indicated that they did for health systems and single facilities, respectively.

**Figure 2:**
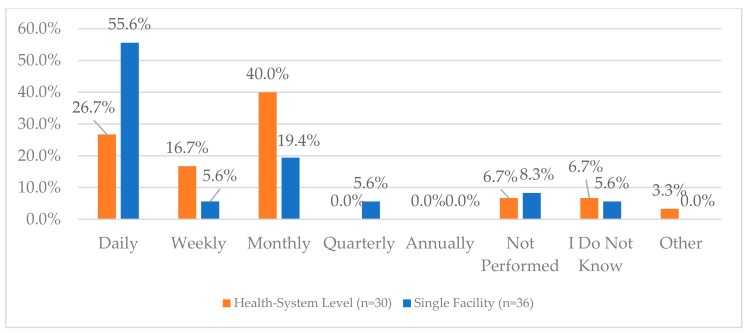
“How often do you audit invoices against the receiving purchase order for CS?”.

**Figure 3:**
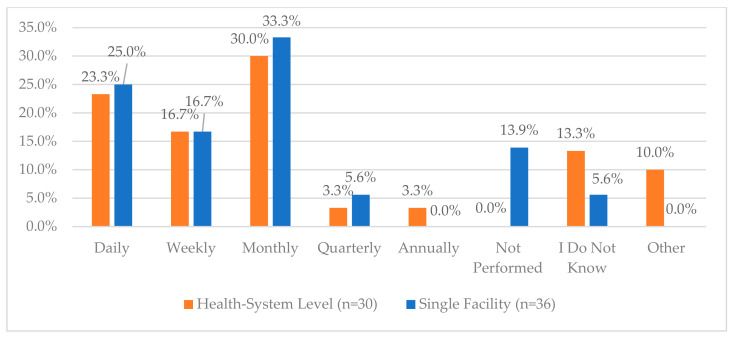
“How often do you audit invoices against the controlled substance vault records?”.

**Figure 4:**
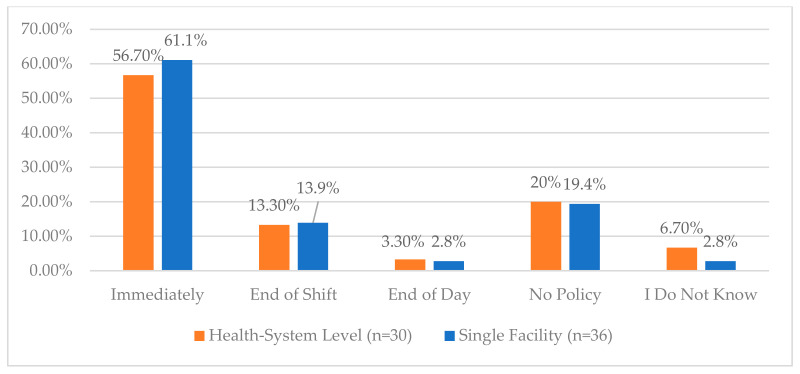
“Per your organization policy and procedure, how soon after receiving controlled substances inventory in the pharmacy should it be entered into the vault?”.

**Figure 5:**
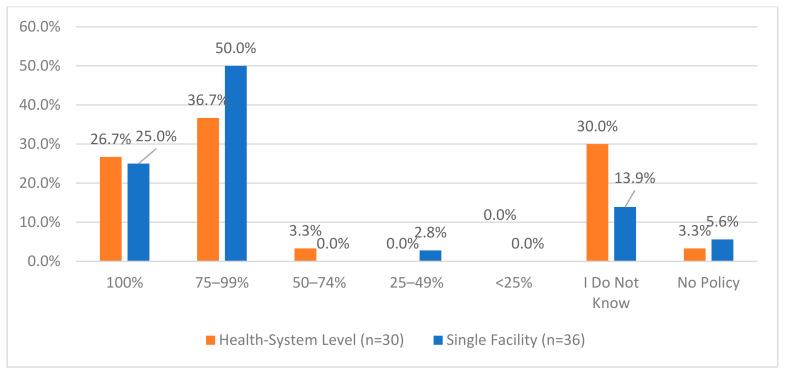
“Approximately what percent of the time is your staff able to meet the policy and procedure for time from controlled substances inventory receipt to entering into the vault?”.

One area of the lowest reported compliance was related to a process for routinely monitoring the effectiveness of Periodic Automated Replenishment (PAR) levels of CS medications and making the appropriate modifications—this was reported to be 60% and 58.3% for health systems and single facilities, respectively. The lowest reported compliance for single facilities was regarding working with their wholesaler to validate CS orders (52.8%) while this was 80% for health systems. Additionally, when asked about the frequency of auditing invoices against the receiving CS purchase order, 55.6% of single facilities reported that this was performed daily, while only 26.7% of health systems reported the same frequency; the most common frequency for health systems was monthly (40%) (Figure 2). There were some health systems and single facilities that reported that these audits were not performed (6.7% and 5.6%, respectively). The complete list of questions and responses can be viewed in Figure 1, Figure 2, Figure 3, Figure 4 and Figure 5.

### 3.3. Controlled Substance Storage, Packaging and Compounding

**Figure 6:**
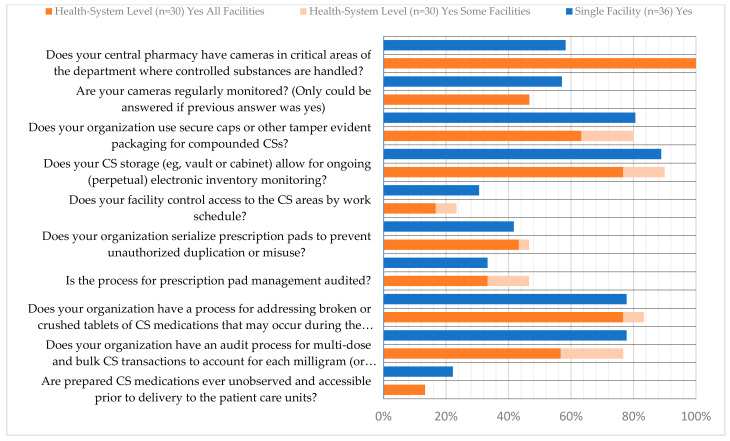
Storage, Packaging and Compounding Survey Responses.

**Figure 7:**
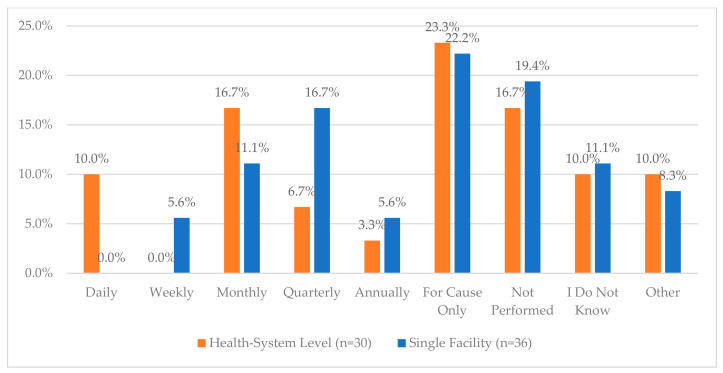
“How often are key and access codes audited and updated?”.

**Figure 8:**
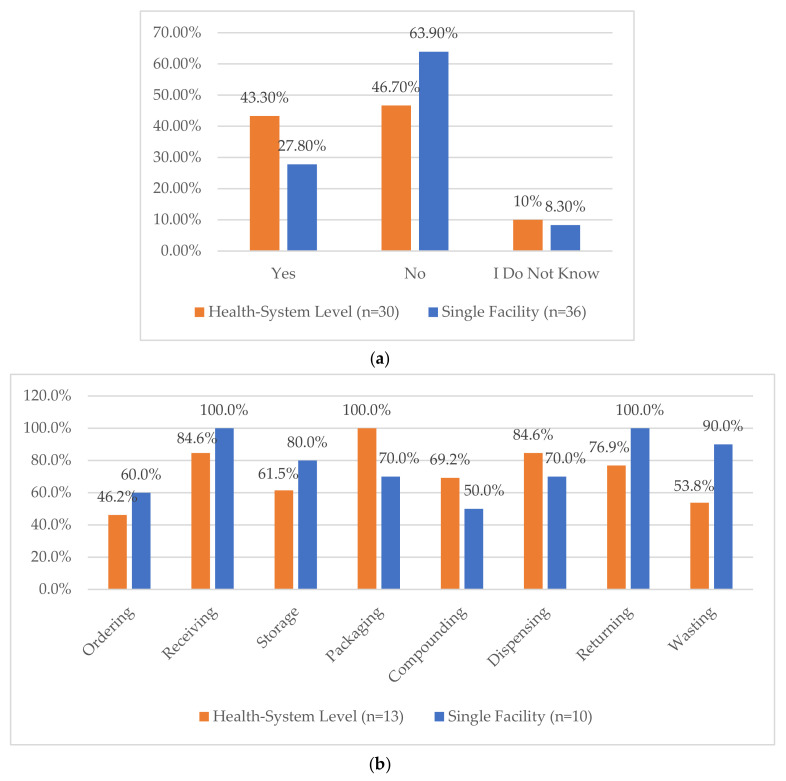
(**a**) “Does your facility rotate roles associated with inventory management?”. (**b**) “If Yes to [previous question], Which roles are regularly rotated? (select all that apply)”.

The nine survey questions and responses regarding the process for storage, packaging, and compounding processes are provided in Figure 6. The area with the lowest reported compliance was related to controlling access to CS areas by work schedule, with only 23.7% and 30.6% of health systems and single facilities, respectively, meeting the recommendations. Similarly, key and access auditing practices varied significantly across both health systems and single facilities with over 40% indicating that it was not performed or only performed for cause (Figure 7). Further, when asked whether a facility regularly rotated roles associated with inventory management, 43.3% of health systems and 27.8% of single facilities indicated that they did. The list of roles that are rotated was also provided (Figure 8a,b).

Regarding the management of prescription pads, single facilities indicated that they serialize prescription pads in 41.7% of responses and that there is an audit process for prescription pad management in 33.3% of responses; for those same questions, both activities were carried out in 46.3% of health systems.

**Figure 9:**
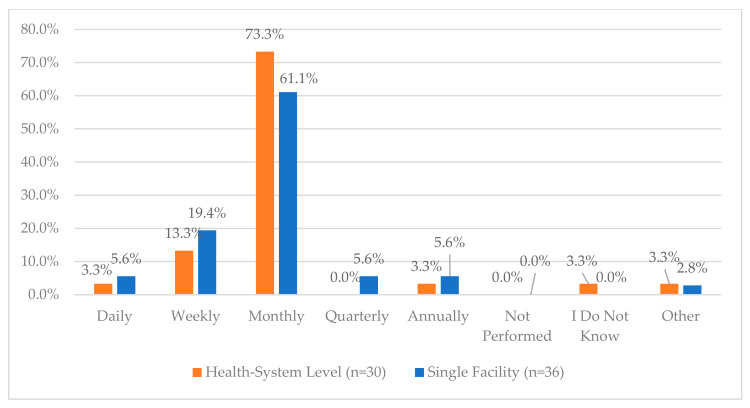
“How often is an audit performed on the controlled substances vault at your facility?”.

The highest area of reported compliance for health systems was the use of cameras in critical areas of the department where CS are handled, with 100% compliance. This was indicated in only 58.3% of single facility responses. For health systems and single facilities, fewer respondents indicated that those cameras are monitored (46.7% and 57.1%, respectively). One area of higher reported compliance for health systems and single facilities was regarding ongoing electronic inventory monitoring for CS storage (89.7% and 88.9%, respectively). Further, when asked about the frequency of audits performed on CS vault inventory, the majority of health systems and single facilities indicated monthly (73.3% and 61.1%, respectively) (Figure 9).

**Figure 10:**
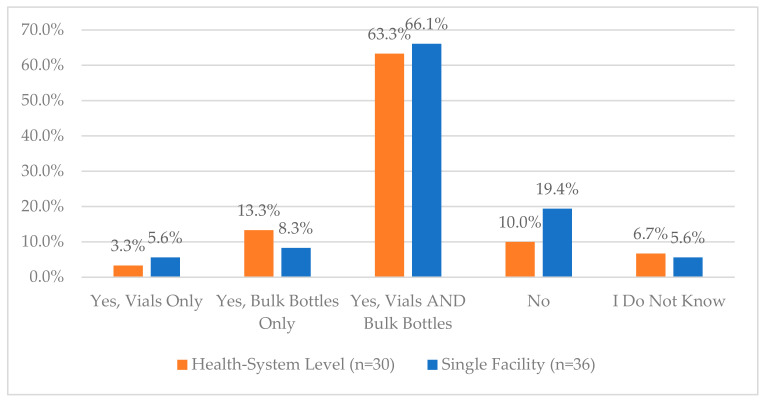
“Does your organization have a process for storing and auditing controlled substance multi-dose vials and bulk bottles when they are not in use?”.

**Figure 11:**
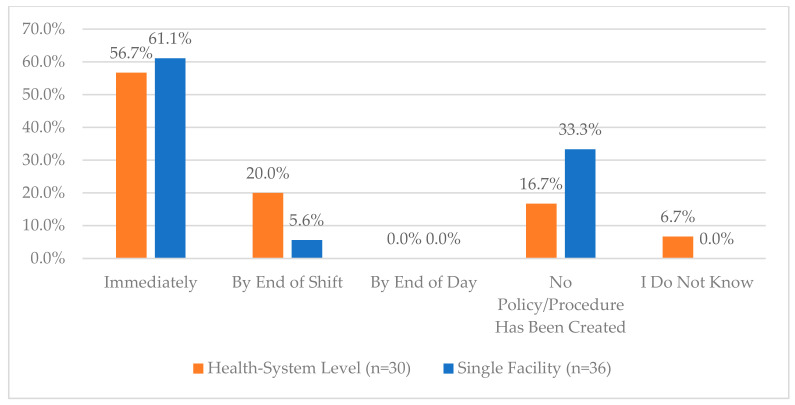
“Per your organization policy or procedure, how soon are repackaged and/or bulk bottles returned to the vault (e.g., time waiting for verification before return to vault)?”.

**Figure 12:**
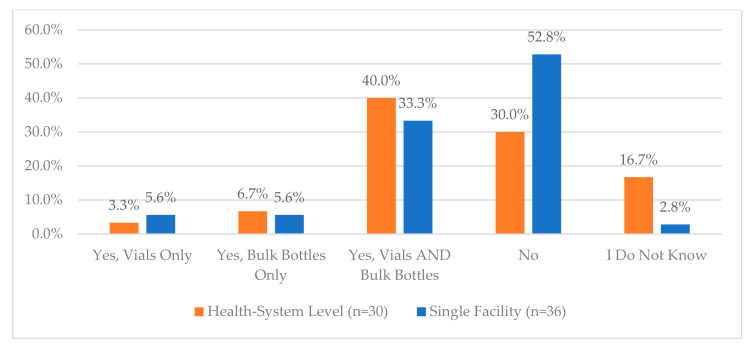
“Does your organization have a policy about how to address over/underfill in vials and bottles of controlled substances?”.

**Figure 13:**
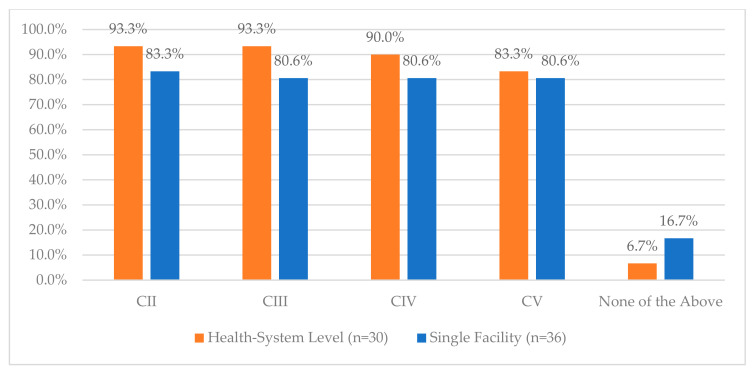
“For which of the following classes does your facility use blind counts in vaults for controlled substance medications? (select all that apply)”.

**Figure 14:**
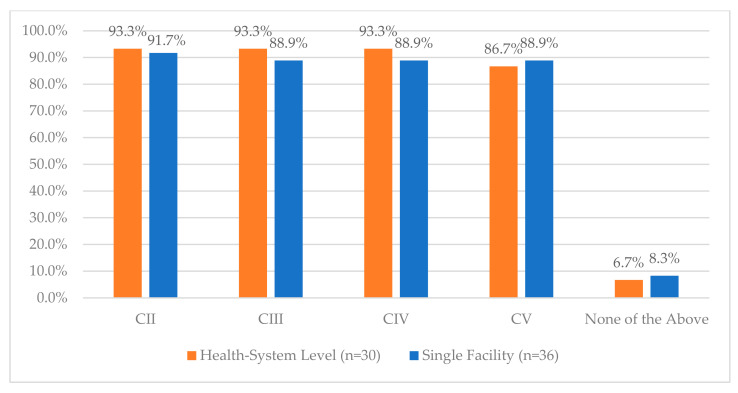
“For which of the following classes does your facility use blind counts in automated dispensing cabinets (ADCs) for controlled substance medications? (select all that apply)”.

Other questions regarding the use of tamper-evident packaging, the process for addressing damaged CS medications, and managing bulk and multi-dose CS can be viewed in Figure 6, Figure 10, Figure 11 and Figure 12. Additional questions regarding the use of “blind” inventory counts for the CS vault and ADCs are in Figure 13 and Figure 14.

### 3.4. Controlled Substance Dispensing

**Figure 15:**
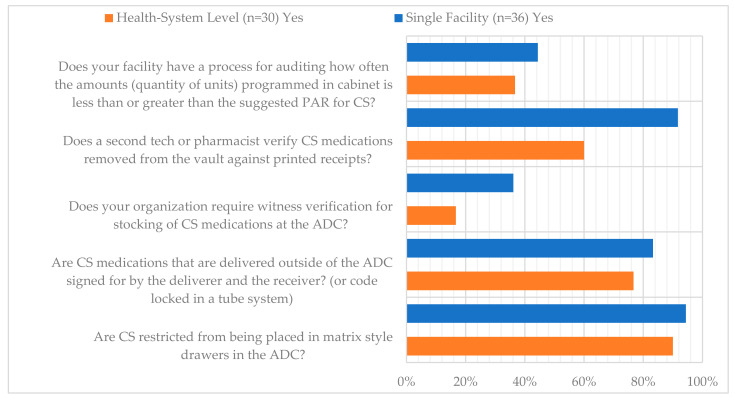
Dispensing Survey Questions.

**Figure 16:**
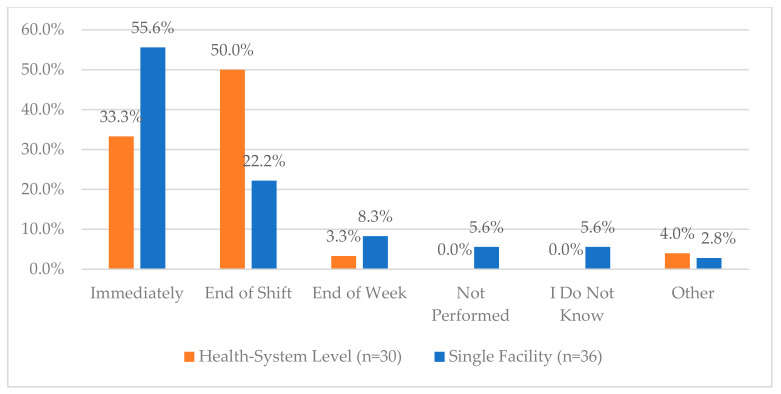
“How soon does your organization require investigation of occurrences of controlled substance inventory count adjustments and discrepancies made by pharmacy personnel? (Either in ADCs or Vault)”.

The six survey questions and responses regarding CS dispensing practices are listed in Figure 15. The highest area of reported compliance was for health systems (90%) and single facilities (94.4%) regarding CS being restricted from being placed in matrix-style drawers in automated dispensing cabinets (ADCs). When CS are delivered outside of the ADC (e.g., patient-specific), they are signed for by both the deliverer and receiver 77% and 83.3% of the time for health systems and single facilities, respectively. The largest difference in practices in respondent type appeared regarding a process for a second verification for CS removed from the vault compared to printed receipts (60% of health systems compared to 91.7% of single facilities). The two areas of lowest reported compliance for each respondent type were regarding witness verification for stocking of CS at the ADC (17% of health systems and 36.1% of single facilities) and auditing how often the amount of CS programmed in the ADC is less than suggested PAR (37% for health systems and 44.4% for single facilities). Additional survey responses regarding CS dispensing and inventory discrepancies are available in Figure 16.

### 3.5. Controlled Substance Waste, Return and Disposal

**Figure 17:**
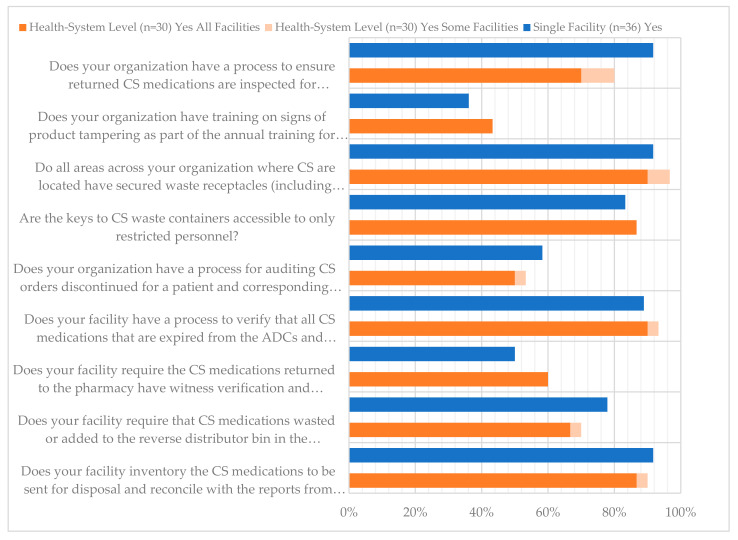
Waste, Return and Disposal Survey Responses.

**Figure 18:**
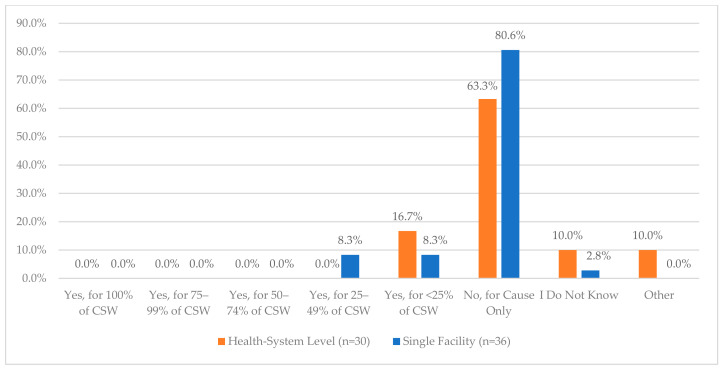
“Does your organization use assay technology to audit wasted drugs?”.

**Figure 19:**
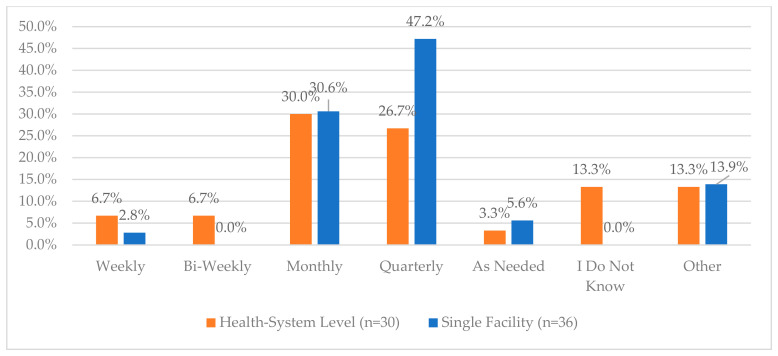
“How often is your reverse distributor bin emptied?”.

**Figure 20:**
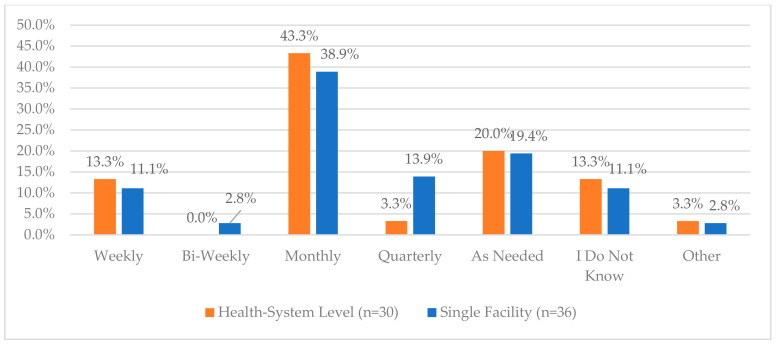
“How often is the inventory in the controlled substance reverse distributor bin audited (e.g., prior to being sent to reverse distributor)?”.

The nine survey questions and responses regarding CS waste return and disposal processes are listed in Figure 17. The lowest reported compliance rates in this category for both health systems and single facilities was regarding training of staff on signs of product tampering as part of annual training (43.3% and 36.1%, respectively). However, most health systems and single facilities indicated that they had a process for ensuring that CS returned to the pharmacy were inspected for tampering (80% and 91.7%, respectively). When asked whether there was a process for auditing CS orders that were discontinued for a patient and a corresponding return to the CS vault, 53.3% and 58.3% indicated “yes” for health systems and single facilities, respectively. Similarly, for CS medications returned to the pharmacy, 60% and 50% indicated that witness verification and signature were required in the pharmacy for health systems and single facilities, respectively. For questions related to CS waste processes, at least 50% of respondents reported compliance for each. For example, there was over 90% reported compliance for both respondent types regarding the presence of secured waste receptacles in all areas where CS are stored. There was also close to 90% reported compliance for a process to verify that all expired meds are in fact expired and placed in the appropriate waste bin. Further, there was at least 90% reported compliance with an inventory of CS medications to be sent for disposal and reconciliation with reverse distributor reports. Additional survey responses regarding CS waste, return, and disposal are available in Figure 18, Figure 19 and Figure 20.

## 4. Discussion

The survey revealed considerable variation in recommended practices for preventing and detecting controlled substance diversion. Larger institutions were more likely to implement these practices compared to smaller ones, which was previously noted by McClure et al. [19]. The highest area of reported compliance for health systems was the installation of cameras in critical areas where CS are handled (100%), whereas this was indicated by only 58.3% of single facility respondents. However, fewer respondents indicated that these cameras are monitored, with 46.7% and 57.1% for health systems and single facilities, respectively.

One of the lowest reported compliance areas was related to routinely monitoring the effectiveness of PAR levels of controlled substances (CS) medications and making appropriate modifications. This was reported to be 60% and 58.3% for health systems and single facilities, respectively. The lowest reported compliance for single facilities was regarding working with their wholesaler to validate CS orders (52.8%), while this was 80% for health systems. Additionally, the frequency of auditing invoices against the receiving CS purchase order varied, with 55.6% of single facilities performing this daily, compared to only 26.7% of health systems, where the most common frequency was monthly (40%).

Tampering continues to be a risk that demands further attention from pharmacy leaders, with less than half of the respondents reporting training in this area. This highlights the need for ongoing education and training programs to ensure that pharmacy staff are well-equipped to handle potential diversion scenarios. The lack of adequate training and monitoring increases the risk of undetected diversion, leading to potential patient harm and legal liabilities for healthcare institutions. Addressing these gaps is important, as failure to do so could result in significant safety threats and financial losses due to diversion-related incidents.

Notably, a small number of respondents reported not using blind counts, and the auditing of drug waste for accuracy is rare. Additionally, nearly 40% of respondents indicated that access codes were either updated “for cause” or not performed at all, demonstrating a significant area of opportunity for improvement. These risk factors could complicate the identification of potential diverters if CS discrepancies are not caught with the immediate subsequent inaccurate inventory count (i.e., blind counts) or due to an unnecessarily large volume of users for a given access code.

There are several strengths of CS management highlighted by the data. Approximately 71.2% of respondents indicated that they utilize drug diversion software and have a dedicated drug diversion surveillance team. Reported compliance with consistent segregation of duties for ordering and receiving processes was high, with 86.7% and 88.9% for health systems and single facilities, respectively. While 53.4% and 63.9% of health systems and single facilities, respectively, indicated that they had such processes in place.

Although our survey did not specifically assess automated dispensing cabinets (ADCs), their use by many respondents aligns with best practices. ADCs play an integral role in controlled substance diversion prevention by providing secure storage, limiting unauthorized access, and generating detailed audit records [3]. Future research could explore optimizing ADC functionality and integration with advanced technologies to address evolving challenges.

There are limitations to this study that should be acknowledged. First, we received 66 survey responses which provides a limited representation of all hospitals and health systems across the US. While the response rate for this survey was low, it is comparable to response rates in similar studies targeting hospital pharmacy leadership, which typically fall in the low 20% range [22,23]. Our initial response rate was 20%; however, after excluding a significant portion of incomplete responses, the final rate was reduced to 14%. With that said, there was representation from different facility types and diverse geographic regions. Second, the research was conducted through a self-reporting survey which introduces subjectivity and potential bias based on respondent interpretation; organizations may consider a formal audit of their practices for more objective measures of compliance with best practices. Third, given the anonymity of the survey, we were unable to ensure that there were no facilities or organizations duplicated in the data. Finally, for the health system level questions, we were not able to determine to what extent their individual facilities have implemented certain practices. However, the goal of the study was not to measure compliance of facilities with drug diversion practices, but rather highlight potential areas of risk and where the healthcare and industry community can further develop strategies to address drug diversion risk within hospital pharmacies.

Despite the establishment of guidelines in 2022 aimed at preventing the diversion of controlled substances, significant gaps remain in addressing potential chain-of-custody issues and auditing practices (e.g., invoices versus orders). These findings may be attributed to the lack of adequate surveillance coverage of the manual tasks traditionally required for auditing, such as ordering and receiving inventory assessments. Overall, based on the survey findings, there remains a need for the development of more advanced surveillance systems, even in hospitals that already deploy automation and technology, which can support health systems in addressing pharmacy-specific gaps.

## 5. Conclusions

This research includes insights from U.S. health systems and reveals gaps in current practices of drug diversion surveillance within hospital pharmacies. While many health systems and single facilities report strategies and policies that have been established to prevent controlled substance diversion, variability in implementation and monitoring persists. Health systems should actively address these gaps in drug diversion risk points in the hospital pharmacy, and implement advanced monitoring, auditing, and staff training to effectively strengthen diversion prevention efforts.

## Figures and Tables

**Table 1:** Respondent Demographics.

Respondent Demographics	Number	Percent
Number of Respondents	66	100%
Role/Job Title (Select All Roles That Apply)		
Staff/Operations Pharmacist	1	1.5%
Clinical Pharmacist	4	6.1%
Pharmacy Manager/Supervisor	11	16.7%
Director of Pharmacy	25	37.9%
Chief Pharmacy Officer/Vice President of Pharmacy	2	3.0%
Drug Diversion Team Member	7	10.6%
Drug Diversion Lead/Director	19	28.8%
Safety, Quality, or Compliance Officer	4	6.1%
Pharmacy Informaticist/Analyst	4	6.1%
Geography (US Division) *^		
New England	5	7.6%
Middle Atlantic	4	6.1%
East North Central	13	19.7%
West North Central	6	9.1%
South Atlantic	16	24.2%
East South Central	4	6.1%
West South Central	7	10.6%
Mountain	3	4.5%
Pacific	6	9.1%
Single Facility or IDN		
Single Facility	36	54.5%
System of Facilities	30	45.5%
Type of Facility (If Single Facility, n = 36)		
Academic Medical Center	6	16.7%
Community Hospital	26	39.4%
Government Hospital	2	5.6%
Long-Term Care Facility	1	2.8%
Surgical Hospital	1	2.8%
Bed Size (If Single Facility, n = 36)		
<150	18	50.0%
150–300	9	25.0%
301–500	8	22.2%
>500	1	2.8%
Diversion System		
Facility(ies) Utilizes Drug Diversion Software	47	71.2%
Facility(ies) Have a Dedicated Drug Diversion Surveillance Team	47	71.2%

* 1 Response: Not U.S. ^ 1 Blank Response 31 States.

**Table 2:** “Who in your organization authorizes purchase orders of controlled substance medications? Please select all that apply.”.

Responses	Health-System Level (n = 30)	Single Facility (n = 36)
Pharmacy Buyer	15 (50.0%)	20 (55.6%)
Pharmacist Manager	26 (86.7%)	29 (80.6%)
Pharmacist Non-Manager	12 (40.0%)	12 (33.3%)
Pharmacist Director	0 (0.0%)	4 (11.1%)
Pharmacy Technician Manager	2 (6.7%)	2 (5.6%)
Pharmacy Technician Non-Manager	5 (16.7%)	1 (2.8%)
Other	0 (0.0%)	1 (2.8%)

Other: State Auditor.

## Data Availability

The original contributions presented in the study are included in the article, further inquiries can be directed to the corresponding author/s.

## Appendix A. Survey Questions

1. Which most closely matches your role (select more than one if necessary):
  ▯ Staff/operations pharmacist
  ▯ Clinical pharmacist
  ▯ Pharmacy manager/supervisor
  ▯ Director of Pharmacy
  ▯ CPO/VP of Pharmacy
  ▯ Drug Diversion Team Member
  ▯ Drug Diversion Lead/Director
  ▯ Safety, Quality, or Compliance Officer
  ▯ Pharmacy Informaticist/Analyst
  ▯ Other:
2. Do you perform any drug diversion surveillance activities as part of your role?
  ▯ Yes
  ▯ No
3. In which state do you primarily work?
4. Does your specific drug diversion surveillance role cover one facility or a system of facilities?
  ▯ System of facilities (e.g., IDN, system level role)
  ▯ Single facility
5. Which most closely matches your facility type:
  ▯ Academic medical center
  ▯ Community hospital
  ▯ Government hospital
  ▯ Long-term care facility
  ▯ Retail pharmacy
  ▯ Industry pharmacy
  ▯ Ambulatory care pharmacy
  ▯ Other:
6. What is your hospital bed size?
  ▯ <150
  ▯ 150–300
  ▯ 300–500
  ▯ 500

Note: the remaining questions were asked at the facility level or system level based on the response to question #4 above. If answering for the system level, the yes/no responses provided the following choices: “yes, for all facilities; yes, for some facilities, no, I do not know.” For the facility level, the choices were “yes, no, I do not know.”

**Procurement**

Please select the most appropriate answer for the following questions:

Yes—for all facilities, Yes—for some facilities, No, I do not know

1. Do you actively work with your wholesaler to validate controlled substance orders? (CS)
2. Do you assess the appropriateness of CS order quantity based on demand (e.g., medication orders and use)?
3. Do you assess for significant changes in ordering patterns of CS over time, including changes in quantity or frequency?
4. Does your organization have a process for routinely monitoring the effectiveness of PAR levels of CS medications and modifying PARs to meet demand fluctuations?
5. Does your organization provide a unique login for each user who can create purchase orders?
6. Does your organization have a policy to purchase unit doses (instead of bulk) of controlled substances when available?

Who in your organization authorizes purchase orders of controlled substance medications? Please select all that apply.

▯ Pharmacy Buyer
▯ Pharmacist Manager
▯ Pharmacist Non-Manager
▯ Pharmacy Technician Manager
▯ Pharmacy Technician Non-Manager
▯ Other

**Receiving**

Please select the most appropriate answer for the following questions:

Yes—for all facilities, Yes—for some facilities, No, I do not know

1. Does your controlled substances ordering and receiving process ensure consistent segregation of duties (e.g., separate team members performing ordering and receiving functions)?
2. Do you have a process for auditing your segregation of duties (person ordering against the person receiving)?

How often do you audit invoices against the receiving purchase order for controlled substances?

▯ Not performed
▯ Daily

How often do you audit invoices against the receiving purchase order for controlled substances?

▯ Not performed
▯ Daily
▯ Weekly
▯ Monthly
▯ Quarterly
▯ Annually
▯ I do not know
▯ Other (add frequency below)

How often do you audit invoices against the controlled substance vault records?

▯ Not performed
▯ Daily
▯ Weekly
▯ Monthly
▯ Quarterly
▯ Annually
▯ I do not know
▯ Other (add frequency below)

Per your organization’s policy and procedure, how soon after receiving controlled substances inventory in the pharmacy should it be entered into the vault?

▯ No policy
▯ Immediately
▯ End of shift
▯ End of day
▯ I do not know
▯ Other (add frequency below)

Approximately what percent of the time is your staff able to meet the policy and procedure for time from controlled substances inventory receipt to entering into the vault?

▯ 100%
▯ 75–99%
▯ 50–74%
▯ 25–49%
▯ <25%
▯ I do not know
▯ Other (add frequency below)

**Storage**

Does your facility rotate roles associated with inventory management?

▯ Yes
▯ No
▯ I do not know

Which roles are regularly rotated? (select all that apply)

▯ Ordering
▯ Receiving
▯ Storage
▯ Packaging
▯ Compounding
▯ Dispensing
▯ Returning
▯ Wasting

How often is an audit performed on the controlled substances vault at your facility?

▯ Not Performed
▯ Daily
▯ Weekly
▯ Monthly
▯ Quarterly
▯ Annually
▯ I do not know
▯ Other (enter frequency below)

Does your central pharmacy have cameras in critical areas of the department where controlled substances are handled?

▯ Yes
▯ No
▯ I do not know

Are your cameras regularly monitored?

▯ Yes
▯ No
▯ I do not know

For which of the following classes does your facility use blind counts in vaults for controlled substance medications? (select all that apply)

▯ CII
▯ CIII
▯ CIV
▯ CV
▯ None of the above

For which of the following classes does your facility use blind counts in automated dispensing cabinets (ADCs) for controlled substance medications? (select all that apply)

▯ CII
▯ CIII
▯ CIV
▯ CV
▯ None of the above

Does your organization have a process for storing and auditing controlled substance multi-dose vials and bulk bottles when they are not in use?

▯ Yes, vials only
▯ Yes, bulk bottles only
▯ Yes, vials and bulk bottles
▯ No
▯ I do not know

How often are key and access codes audited and updated?

▯ Not Performed
▯ Daily
▯ Weekly
▯ Monthly
▯ Quarterly
▯ Annually
▯ For cause only
▯ I do not know
▯ Other (enter frequency below)

Please select the most appropriate answer for the following questions:

Yes—for all facilities, Yes—for some facilities, No, I do not know

1. Does your organization use secure caps or other tamper-evident packaging for compounded controlled substances (CS)?
2. Does your CS storage (e.g., vault or cabinet) allow for ongoing (perpetual) electronic inventory monitoring?
3. Does your facility control access to the CS areas by work schedule?
4. Does your organization serialize prescription pads to prevent unauthorized duplication or misuse?
5. Is the process for prescription pad management audited?

**Packaging/Compounding**

Please select the most appropriate answer for the following questions:

Yes—for all facilities, Yes—for some facilities, No, I do not know

1. Does your organization have a process for addressing broken or crushed tablets of controlled substance (CS) medications that may occur during the repackaging process?
2. Does your organization have an audit process for multi-dose and bulk CS transactions to account for each milligram (or unit)?

Per your organization’s policy or procedure, how soon are repackaged and/or bulk bottles returned to the vault (e.g., time waiting for verification before return to the vault)?

▯ No policy/procedure has been created
▯ Immediately
▯ By end of shift
▯ By end of day
▯ Other
▯ I do not know

Does your organization have a policy about how to address over/underfill in vials and bottles of controlled substances?

▯ Yes, vials only
▯ Yes, Bulk bottles only
▯ Yes, for vials and bulk bottles
▯ No
▯ I do not know

**Dispensing**

Please select the most appropriate answer for the following questions:

Yes—for all facilities, Yes—for some facilities, No, I do not know

1. Are prepared controlled substance (CS) medications ever unobserved and accessible prior to delivery to the patient care units?
2. Does your facility have a process for auditing how often the amounts (quantity of units) programmed in the cabinet are less than or greater than the suggested PAR for CS?
3. Does a second tech or pharmacist verify CS medications removed from the vault against printed receipts?
4. Does your organization require witness verification for stocking of controlled substance (CS) medications at the ADC?
5. Are CS medications that are delivered outside of the ADC signed for by the deliverer and the receiver? (or code-locked in a tube system)
6. Are CS restricted from being placed in matrix-style drawers in the ADC?

How soon does your organization require an investigation of occurrences of controlled substance inventory count adjustments and discrepancies made by pharmacy personnel? (Either in ADCs or Vault)

▯ Not performed
▯ Immediately
▯ End of shift
▯ End of week
▯ I do not know
▯ Other

**Waste, Return, Disposal**

Please select the most appropriate answer for the following questions:

Yes—for all facilities, Yes—for some facilities, No, I do not know

1. Does your organization have a process to ensure returned controlled substance (CS) medications are inspected for tampering prior to returning to circulation for potential use?
2. Does your organization have training on signs of product tampering as part of the annual training for individuals?
3. Do all areas across your organization where CS are located have secured waste receptacles (including pharmacy department space)?
4. Are the keys to CS waste containers accessible to only restricted personnel?
5. Does your organization have a process for auditing controlled substance (CS) orders discontinued for a patient and corresponding return to the controlled substance vault?
6. Does your facility have a process to verify that all CS medications that are expired from the ADCs and vault are indeed expired and placed in the appropriate waste/reverse distributor bin?
7. Does your facility require the CS medications returned to the pharmacy to have witness verification and signature?
8. Does your facility require that CS medications wasted or added to the reverse distributor bin in the pharmacy have witness verification and signature?
9. Does your facility inventory the CS medications to be sent for disposal and reconcile with the reports from the reverse distributor?

Does your organization use assay technology to audit wasted drugs?

▯ No, for cause only
▯ Yes, for 100% of controlled substance waste
▯ Yes, for 75–99% of controlled substance waste
▯ Yes, for 50–74% of controlled substance waste
▯ Yes, for 25–49% of controlled substance waste
▯ Yes, for <25% of controlled substance waste
▯ Other
▯ I do not know

How often is your reverse distributor bin emptied?

▯ Weekly
▯ Bi-weekly
▯ Monthly
▯ Quarterly
▯ As needed
▯ Other
▯ I do not know

How often is the inventory in the controlled substance reverse distributor bin audited (e.g., prior to being sent to the reverse distributor)?

▯ Weekly
▯ Bi-weekly
▯ Monthly
▯ Quarterly
▯ As needed
▯ Other
▯ I do not know

**Resources**

Please select the most appropriate answer for the following questions:

Yes—for all facilities, Yes—for some facilities, No, I do not know

1. Does your facility have a dedicated drug diversion surveillance team?
2. Does your facility utilize drug diversion software?

How frequently are staff trained on drug diversion policies and identification?

▯ Once
▯ Quarterly
▯ Annually
▯ Other
▯ I do not know
▯ No training
